# Large-vessel-occlusion in patients with previous ischemic stroke: an analysis of adherence to secondary preventive medication for different etiologies

**DOI:** 10.1186/s42466-023-00247-7

**Published:** 2023-05-25

**Authors:** Konstantin Kohlhase, Jan Hendrik Schäfer, Lisa Marie Tako, Laurent M. Willems, Elke Hattingen, Ferdinand O. Bohmann, Christian Grefkes, Felix Rosenow, Adam Strzelczyk

**Affiliations:** 1 Department of Neurology and Epilepsy Center Frankfurt Rhine-Main, University Hospital and Goethe University Frankfurt, Frankfurt am Main, Germany; 2 LOEWE Center for Personalized and Translational Epilepsy Research (CePTER), Goethe-University Frankfurt, Frankfurt am Main, Germany; 3 Institute of Neuroradiology, University Hospital and Goethe University Frankfurt, Frankfurt am Main, Germany

**Keywords:** Secondary prophylaxis, Large vessel occlusion, Stroke unit

## Abstract

**Background:**

Large vessel occlusion (LVO) is a severe condition that carries a high risk of morbidity and mortality, underscoring the importance of effective prevention strategies. This retrospective study aimed to analyze the intake of preventive medication at the time of hospitalization in a cohort of recurrent stroke patients presenting with acute LVO.

**Methods:**

The study assessed the intake of either platelet aggregation inhibitors (PAI), oral anticoagulants (OAC) or statins at admission in patients with recurrent stroke and correlated it with the final classification of LVO. The frequency of those secondary preventive medication in recurrent stroke patients was defined as primary endpoint. The Modified Rankin Scale (mRS) at discharge was used as a functional outcome and defined as a secondary outcome measure.

**Results:**

This study included 866 patients who were treated for LVO between 2016 and 2020, of whom 160 (18.5%) had a recurrent ischemic stroke. OAC (25.6% vs. 14.1%, p < 0.01), PAI (50.0% vs. 26.0%, p < 0.01), or statin therapy (50.6% vs. 20.8%, p < 0.01) at admission were significantly more frequent in recurrent stroke patients compared to patients with a first-time stroke. Concerning LVO etiology in recurrent stroke patients, OAC at admission was taken in 46.8% of cardioembolic LVO, whereas PAI and statin at admission in macroangiopathic LVO were administered to 40.0%; neither PAI nor OAC was taken in 26.0%, 28.3%, and 31.6% of cardioembolic, macroangiopathic, or cryptogenic strokes, respectively. Regardless of stroke recurrence or etiology, there was an increase in mRS at discharge.

**Conclusions:**

Despite high-quality healthcare, this study suggested a significant proportion of patients with recurrent stroke who were either non-adherent or insufficiently adherent to secondary preventive medication. Given the disability associated with LVO, improving patients’ medication adherence and identifying unknown stroke causes are crucial for effective prevention strategies.

## Background

In 2019, stroke was responsible for 12.2 million new cases and 6.55 million deaths worldwide, making it the second leading cause of death and the third leading cause of disability []. Although mortality rates and age-specific incidence have declined in recent decades due to improved stroke care in highly developed countries, the absolute number of stroke cases has continued to rise due to the aging of the population [1]. Following a stroke, approximately one-third of patients die within the first 30 days, and up to two-thirds within 5 years. Additionally, 37–45.3% of stroke survivors remain functionally dependent (with a modified Rankin Scale [mRS] score of > 2) one year after the stroke [2–4]. The high incidence and impact of stroke on patient outcomes also translate to significant costs for a society’s healthcare system—these average to $30,633 per year in high-income countries [5]. In Europe, the costs of stroke care comprised 1.7% of total health care spending in 2017 [6]. Demographic changes and advancements in stroke care have led to a shift towards higher morbidity rates and increased burden on healthcare systems, despite a decrease in mortality rates [7]. Therefore, effective primary and secondary prevention are crucial for reducing the incidence of first and recurrent strokes, which can range from 9 to 15% in the first year [8, 9]. In addition to managing common cardiovascular risk factors such as diabetes mellitus, arterial hypertension, and obesity, post-stroke care commonly includes the use of antiplatelet drugs, statin therapy, and oral anticoagulants [9]. Among the various types of ischemic stroke, the occurrence of large vessel occlusion (LVO) is especially concerning due to its high risk of mortality and morbidity, and therefore, it holds a significant status in stroke prevention [10].

The objective of this study was to assess the adherence to guideline-based medical secondary stroke prophylaxis (including platelet aggregation inhibitors (PAI), oral anticoagulants (OAC), and statin therapy) in patients with recurrent ischemic stroke and acute LVO compared to a cohort with first-time ischemic stroke.

## Methods

In this study, data from all patients who received treatment for an acute ischemic stroke caused by an LVO at the University Hospital Frankfurt between 2016 and 2020 were retrospectively analyzed. The study was approved by the local ethics committee of the Goethe University Frankfurt (IRB#: 19–285), and data on the occurrence of acute epileptic seizures have been previously published [11, 12]. The STROBE (Strengthening the Reporting of Observational Studies in Epidemiology) guidelines were closely followed [13]. Given the retrospective design, written informed consent from the patients was not required.

### Data collection

Ischemic stroke was diagnosed according to the International Statistical Classification of Diseases and Related Health Problems, 10th Revision [14]. LVO was defined as occlusion of the internal carotid artery, middle cerebral artery (including segments M1 and M2), or basilar artery, and was further divided into anterior (internal carotid artery, middle cerebral artery) or posterior (basilar artery) circulation. A diagnosis of LVO was established through imaging techniques such as CT or MR angiography, or by identifying infarct demarcation attributable to a proximal vessel occlusion. Data on the following variables were collected: age, sex, NIHSS score upon admission and discharge, modified Rankin Scale (mRS) before admission and at discharge, type of LVO (anterior or posterior circulation), stroke etiology, total cholesterol levels (mg/dl), treatment with mechanical thrombectomy or systemic thrombolysis, arterial hypertension, diabetes mellitus, atrial fibrillation, history of previous ischemic stroke, and treatment with OAC (such as Vitamin K antagonists, factor Xa inhibitors, thrombin inhibitors), PAI (such as platelet aggregation inhibitors like aspirin or adenosine diphosphate [ADP] receptor inhibitors), or statins at admission.

### Endpoints

The primary endpoint of this study was adherence to secondary prophylactic medication at admission for patients with a history of previous ischemic stroke, as recommended by the neurological guidelines of the European Stroke Organisation (ESO) and German Neurological Society (DGN) [9, 15]. The current LVO was classified into different stroke causes, including cardioembolism (e.g. atrial fibrillation), large-artery atherosclerosis (with an intraluminal stenosis of ≥ 50%) or stroke of undetermined etiology (“cryptogenic”) and was extracted from the discharge letter. Cryptogenic stroke was considered in patients without identified stroke cause. In patients with two or more causes, the most probable source was used for classification.

The study evaluated whether the initial secondary prophylactic medication was suitable for effective stroke prevention based on the stroke cause. Feasible medical stroke prevention was considered in patients with large-artery atherosclerosis or cryptogenic stroke by the intake of a PAI (such as aspirin or ADP receptor inhibitors like clopidogrel or ticagrelor) and/or statin therapy at admission. Patients with cardioembolic strokes due to atrial fibrillation were appropriately treated with an OAC such as a factor Xa inhibitor, thrombin inhibitor, or Vitamin K antagonist [9, 15].

The secondary end point of the study was defined as the degree of disability and dependence after stroke, indicated by the difference of mRS prior to admission and at discharge between the subgroups (etiology, stroke history) as well as an individual shift from functional independency (mRS ≤ 2) to dependency in activities of daily life (mRS ≥ 3). Furthermore, the difference in baseline characteristics (age, sex, NIHSS at admission and discharge, stroke etiology, cardiovascular risk factors, emergency treatment with systemic thrombolysis or mechanical thrombectomy, and atrial fibrillation) of recurrent stroke patients, subdivided into patients who received OAC or PAI at admission and those who did not, were compared.

### Statistics

Statistical analyses were performed using IBM SPSS Statistics software version 27.0.1.0 (IBM Corp., Armonk, NY, USA). Differences in patient characteristics between the two groups (with and without previous stroke) were assessed using chi-squared tests for categorical variables (e.g. gender, atrial fibrillation) and Mann-Whitney U tests for ordinal or numeric values (e.g. NIHSS, mRS, age). Differences in stroke etiologies and medication at admission (PAI, OAC, statin) were also evaluated using chi-squared tests. Changes in mRS from admission to discharge were evaluated using Wilcoxon signed-rank tests. The mRS was further dichotomized into mRS ≤ 2 (mostly independent in daily life) and mRS ≥ 3 (dependent in activities of daily life), and changes in mRS category during hospital stay were evaluated using chi-squared tests. P-values were adjusted for multiple testing using the Benjamini-Hochberg false discovery rate method, and a p-value of < 0.05 was considered statistically significant.

## Results

The study evaluated 866 patients with LVO who were treated between 2016 and 2020. Of these patients, 160 (18.5%) had a history of previous ischemic stroke. The mean age ± standard deviation (SD) of patients with a first-time stroke was 70.8 ± 14.1 years, while that of patients with a recurrent stroke was 74.0 ± 11.7 years (p = 0.04). Gender distribution (50.2% and 48.1% female patients experiencing first-time and recurrent strokes, respectively) did not show significant differences (p = 0.66). The anterior circulation was affected in 91.9% and 91.7% (p = 0.91) in patients with and without a previous stroke, respectively. There were significant differences in stroke etiologies between the groups, with more cardioembolic and large-artery stenosis strokes in patients with a recurrent stroke (48.1% vs. 40.5% and 37.5% vs. 29.9%, respectively, p < 0.01). Cryptogenic LVO was more common in patients without a prior stroke (24.9% vs. 11.0%; p < 0.01). Patients without a previous stroke underwent mechanical thrombectomy (MT) and systemic thrombolysis (IVT) more frequently than those with a previous stroke (MT: 54.0% vs. 42.5%, p = 0.019, IVT: 49.2% vs. 35.6%, p < 0.01).

OAC (25.6% vs. 14.1%, p < 0.01), PAI (50.0% vs. 26.0%, p < 0.01), and statin therapy (50.6% vs. 20.8%, p < 0.01) were reported more often in patients with a recurrent stroke. Patients with a recurrent stroke also had a higher prevalence of cardiovascular risk factors, such as arterial hypertension (85.6% vs. 70.9%, p < 0.01), diabetes mellitus (34.4% vs. 17.0%, p < 0.01), and atrial fibrillation (51.9% vs. 38.2%, p < 0.01). NIHSS at admission showed no significant difference between the groups (p = 0.53), whereas a higher NIHSS at discharge was more common in patients with a prior stroke (median NIHSS [Q1-Q3]: 4 [2–12] vs. 3[0–9], p = 0.04). Further details are provided in Table 1.

**Table 1:** Characteristics of patients with large vessel occlusion, subdivided into those with and without prior stroke. For intergroup differences, chi-square test was used for binary variables and Mann-Whitney test for ordinal or numeric scaled variables, with a p-value < 0.05 considered significant and marked in bold. Abbreviations: NIHSS – National Institute of Health Stroke Scale, mRS – modified Rankin Scale

Prior stroke	No = 706	Yes = 160	p-value
Age	70.8 ± 14.1	74.0 ± 11.7	**0.04** (Mann-Whitney-Test)
			Chi-Square
Posterior circulation %positive	62; 8.8%	13; 8.1%	0.84
Anterior circulation %positive	648; 91.7%	147; 91.9%	0.91
Sex %female	355; 50.2%	77; 48.1%	0.66
Died %	133; 18.8%	34; 21.3%	0.6
Etiology			
Cryptogenic	176; 24.9%	19; 11.0%	**< 0.01**
Large-artery sclerosis	211; 29.9%	60; 37.5%	
Cardioembolism	286; 40.5%	77; 48.1%	
Others	33; 4.7%	4; 2.5%	
Mechanical thrombectomy %yes	382; 54.0%	68; 42.5%	**0.019**
Systemic thrombolysis %yes	348; 49.2%	57; 35.6%	**< 0.01**
Oral anticoagulants %yes	100; 14.1%	41; 25.6%	**< 0.01**
Platelet aggregation inhibitor %yes	184; 26.0%	80; 50.0%	**< 0.01**
Statins %yes	147; 20.8%	81; 50.6%	**< 0.01**
Cholesterol ≥ 200 mg/dl at admission %yes	109; 19.9%	18; 14.5%	0.26
Diabetes mellitus %yes	120; 17.0%	55; 34.4%	**< 0.01**
Arterial hypertension %yes	561; 70.9%	137; 85.6%	**< 0.01**
Atrial fibrillation %yes	270; 38.2%	83; 51.9%	**< 0.01**
NIHSS at admission (median)	13 (6–18)	12 (6–17)	0.53 (Mann-Whitney-Test)
NIHSS at discharge (median)	3 (0–9)	4 (2–12)	**0.04** (Mann-Whitney-Test)
mRS at admission	0 (0–1)	1 (0–3)	**< 0.01** (Mann-Whitney-Test)
mRS at discharge	4 (1–5)	4 (2–6)	0.06 (Mann-Whitney-Test))

## Analysis by etiology and intake of a secondary prophylactic medication

Among patients with LVO classified as cardioembolic at discharge, 46.8% received OAC at admission, which was more frequent compared to patients with a first-time cardioembolic stroke (27.6%, p < 0.01). PAI with statin (22.1% vs. 10.1%) was significantly more frequent in patients with a recurrent stroke (p < 0.01). A combination of PAI and OAC was present in 6.5% and 2.4% of patients with a recurrent or first-time stroke, respectively, without significant group differences (p = 0.15). Among patients with cardioembolic LVO, 26.0% of patients with a previous ischemic stroke neither took PAI nor OAC at admission, which was, however, less common compared to patients with a first-time cardioembolic stroke (71.5%, p < 0.01).

Patients with LVO due to large-artery stenosis had an overall lower rate of pre-admission OAC, with 6.7% and 4.7% in patients with and without prior stroke (p = 0.66), respectively. PAI alone (25.0% vs. 12.8%, p < 0.01), statin alone (16.7% vs. 6.6%, p < 0.01), and PAI with statin (40.0% vs. 17.5%, p < 0.01) was more frequent in patients with large-artery stenosis and previous ischemic stroke compared to patients with first-time stroke. However, among patients with large-artery stenosis and recurrent stroke, neither PAI nor statin as well as neither PAI nor OAC at admission were received in 18.3% and 28.3%, respectively.

For cryptogenic LVO, there were no significant differences in the frequency of OAC between the subgroups, with an overall low rate of 0.0% and 3.4%, respectively (p = 0.6). In contrast, significant differences were observed between cryptogenic LVO in patients with and without prior stroke concerning PAI alone (31.6% vs. 10.2%, p < 0.01), PAI and statin (36.8% vs. 17.6%, p < 0.01), and no PAI or statin (31.6% vs. 68.8%, p < 0.01) at admission, respectively. In 31.6% of patients with cryptogenic LVO and a previous history of stroke, neither OAC nor PAI was administered (p < 0.01) (Table 2).

**Table 2:** Differences in medical prevention with OAC, PAI, or statin at admission based on the final etiological stroke diagnosis (cardioembolic, macroangiopathic, cryptogenic, or other) and positive history of previous ischemic stroke. Intergroup differences were assessed using a chi-square test, with a significance level of p < 0.05 marked in bold. Abbreviations: PAI – platelet aggregation inhibitor, OAC – oral anticoagulation

	Cardioembolic stroke			Macroangiopathic stroke			Cryptogenic			Other strokes	
	No previous stroke (n = 286)	Recurrent stroke (n = 77)	Chi-square	No previous stroke (n = 211)	Recurrent stroke (n = 60)	Chi-square	No previous stroke (n = 176)	Recurrent stroke (n = 19)	Chi-square	No previous stroke (n = 33)	Recurrent stroke (n = 4)
OAC	N = 79 27.6%	N = 36 46.8%	**< 0.01**	N = 10 4.7%	N = 4 6.7%	0.66	N = 6 3.4%	N = 0 0,0%	0.6	N = 5 15.2%	N = 1 25.0%
PAI	N = 37 12.9%	N = 9 11.7%	**< 0.01**	N = 27 12.8%	N = 15 25.0%	**< 0.01**	N = 18 10.2%	N = 6 31.6%	**< 0.01**	N = 4 12.1%	N = 1 25.0%
Statin alone	N = 29 10.1%	N = 21 27.3%		N = 14 6.6%	N = 10 16.7%		N = 6 3.4%	N = 0 0,0%		N = 0 0,0%	N = 1 25.0%
PAI and Statin	N = 29 10.1%	N = 17 22.1%		N = 37 17.5%	N = 24 40.0%		N = 31 17.6%	N = 7 36.8%		N = 1 3.0%	N = 1 25.0%
No PAI or statin	N = 191 66.8%	N = 30 39.0%		N = 133 63.0%	N = 11 18.3%		N = 121 68.8%	N = 6 31.6%		N = 28 84.8%	N = 12 25.0%
OAC and PAI	N = 7 2.4%	N = 5 6.5%	0.15	N = 1 0.5%	N = 0 0,0%	0.66	N = 0 0,0%	N = 0 0,00%	-	N = 1 3.0%	N = 0 0,0%
No OAC or PAI	N = 148 71.5%	N = 20 26.0%	**< 0.01**	N = 138 65.4%	N = 17 28.3%	**< 0.01**	N = 121 68.8%	N = 6 31.6%	**< 0.01**	N = 24 72.7%	N = 0 0,0%

## Baseline characteristics of patients with recurrent stroke without PAI or OAC at admission

There were no significant differences in baseline characteristics (e.g., age, gender, NIHSS or mRS at admission, p ≥ 0.12), cardiovascular risk factors (e.g., arterial hypertension, diabetes mellitus, hypercholesterolemia, p ≥ 0.23) or presence of atrial fibrillation (p = 0.41) between patients with recurrent stroke who were administered OAC or PAI at admission and those who were not. Patients who received pre-admission OAC or PAI showed a higher frequency of concomitant statin therapy (58.6% vs. 29.5%, respectively; p < 0.01). Furthermore, there was a trend towards a higher mortality in patients who received OAC or PAI at admission (25.0% vs. 11.4%, respectively, p = 0.08), which did not reach the level of significance after correction for multiple testing (Table 3).

**Table 3:** Characteristics of patients with large vessel occlusion and a positive history of ischemic stroke, subdivided into patients with and without the intake of platelet aggregation inhibitors (PAI) or oral anticoagulants (OAC) at admission. For intergroup differences, chi-square test was used for binary variables and Mann-Whitney test for ordinal or numeric scaled variables, a p-value < 0.05 was considered significant and marked in bold. Abbreviations: NIHSS – National Institute of Health Stroke Scale, mRS – modified Rankin Scale

Recurrent stroke	PAI or OAC		
	Yes (n = 116)	No (n = 44)	p-value
Age	74.5 ± 10.8	72.9 ± 13.8	0.69
Posterior circulation %positive	8; 6.9%	5; 11.4%	0.4
Anterior circulation %positive	108; 93.1%	39; 88.6%	0.4
Sex %female	56; 48.3%	21; 47.7%	0.65
Died %	29; 25.0%	5; 11.4%	0.08
Etiology			
Cryptogenic	13; 11.2%	6; 13.6%	0.97
Large-artery sclerosis	43; 37.1%	17; 38.6%	
Cardioembolism	57; 49.1%	20; 45.5%	
Others	3; 2.6%	1; 2.3%	
Mechanical thrombectomy %yes	50; 43.1%	18; 40.9%	0.6
Systemic thrombolysis %yes	43; 37.1%	14; 31.8%	0.47
Statins %yes	68; 58.6%	13; 29.5%	**< 0.01**
Cholesterol ≥ 200 mg/dl at admission %yes	12; 10.3%	6; 13.6%	0.56
Diabetes mellitus %yes	37; 31.9%	18; 40.9%	0.3
Arterial hypertension %yes	102; 87.9%	35; 79.5%	0.23
Atrial fibrillation %yes	58; 50%	25; 56.8%	0.41
NIHSS at admission (median)	12 (7–18)	10 (5–14)	0.12
NIHSS at discharge (median)	3 (1–10)	6 (2–13)	0.23
mRS at admission	1 (1–3)	1 (0–2)	0.48
mRS at discharge	4 (2–6)	4 (2–5)	0.36

## Comparison of mRS scores between different etiologies and recurrent strokes

The mRS scores at admission showed significant differences between patients (total) with and without previous stroke (p < 0.01), as well as in the subgroup analysis of patients with a cardioembolic or large-artery sclerosis origin of stroke (p < 0.01). However, the difference in mRS scores at discharge only showed a trend towards higher scores in patients with a previous ischemic stroke (total), which did not reach significance after correcting for multiple testing (p = 0.06). There was a significant worsening between the mRS scores at admission and at discharge across all etiologies (cryptogenic, large-artery stenosis, cardioembolism) and regardless of stroke history (p < 0.01). However, in the subgroup analysis to determine an individual shift in the mRS group from independent (mRS ≤ 2) to dependent in activities of daily life (mRS ≥ 3), there were no significant differences between patients with and without previous stroke based on their stroke classification (cryptogenic: p = 0.91, large-artery sclerosis: p = 0.17, cardioembolism: p = 0.66) (Table 4).

**Table 4:**
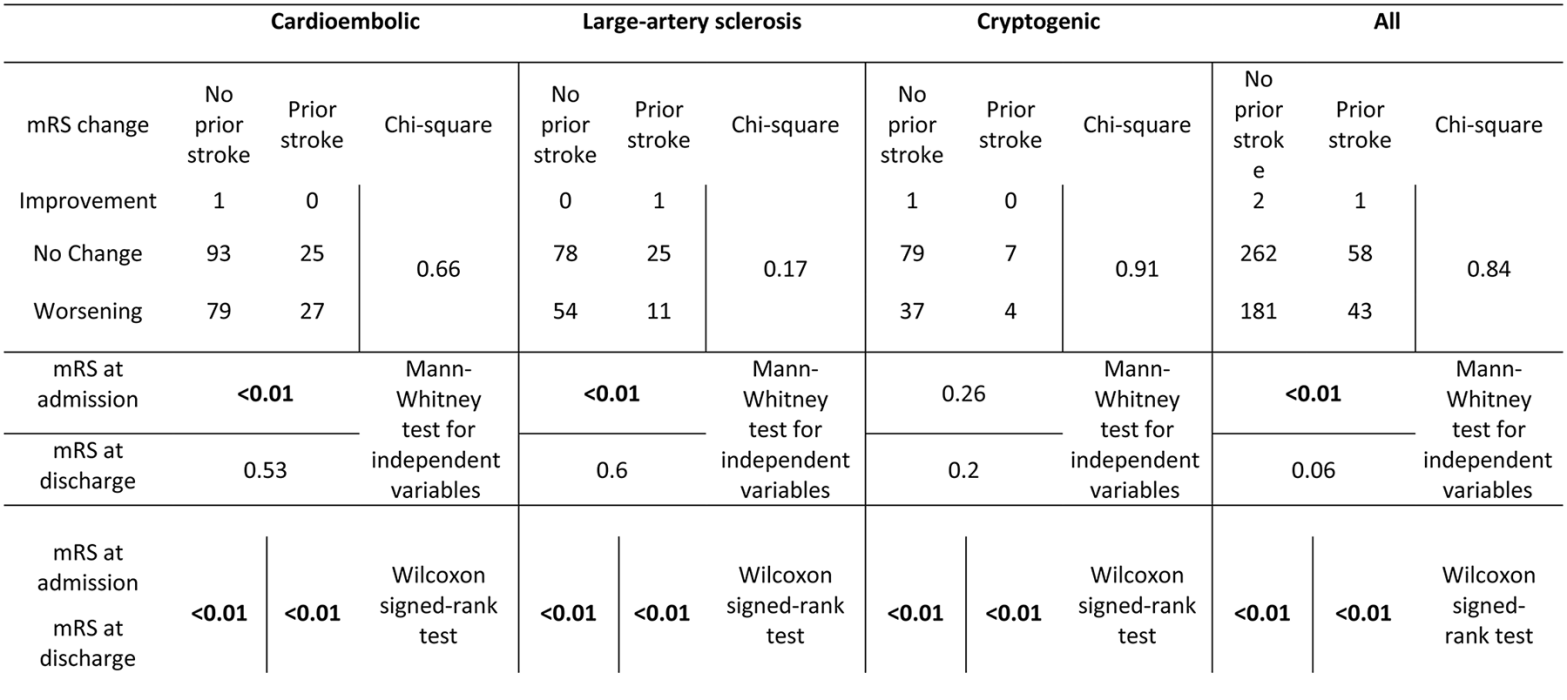
Functional outcome, indicated by mRS before admission and at discharge, among different stroke etiologies (cardioembolic, large-artery sclerosis/macroangiopathic, cryptogenic) and between patients with and without a positive history of previous ischemic stroke. Statistical tests used were based on the scale of variables (ordinal or nominal) and dependence, as indicated. A p-value < 0.05 was considered significant and marked in bold

## Discussion

This study demonstrated that a significant proportion of patients with LVO had a history of recurrent ischemic stroke and differed significantly from patients with first-time stroke in terms of receiving preventive drugs (PAI, statin, or OAC) at admission, etiology of LVO, and presence of cardiovascular risk factors. Specifically, the study found that a high percentage of patients with recurrent stroke were either not taking any preventive antiplatelet or anticoagulant medication or were taking medication that was inadequate for preventing the current LVO.

Among patients with recurrent stroke, a high prevalence of well-known cardiovascular risk factors such as arterial hypertension (85.6%), diabetes mellitus (34.4%), and atrial fibrillation (51.9%) was observed, which was significantly higher than in patients with first-time stroke and also higher than the reported average prevalence of arterial hypertension (28.5%) or diabetes mellitus (10.5%) in high-income countries [16, 17]. The increased prevalence of cardiovascular risk factors among patients with a recurrent ischemic stroke is likely explained by the preselected patient population with a presumably increased cardiovascular risk profile or preexisting noncerebral cardiovascular events/comorbidities (e.g., myocardial infarction, coronary artery disease, peripheral vascular disease). Consistent with this finding, we found overall high rates of pre-admission OAC, PAI or statin therapy in both groups. However, cardiovascular risk factors, as well as the rate of preventive drugs taken, were even more frequent in patients with a recurrent ischemic stroke. Whether the preventive drugs were initiated due to the previous stroke or because of another concomitant cardiovascular disease could not be evaluated thus limiting further differentiation. For stroke causes that require specific secondary stroke prevention measures (e.g., large-artery-stenosis: PAI and statin; cardioembolism: oral anticoagulants), a relevant proportion of recurrent stroke patients did not take a suitable medication at admission that could have effectively reduced the risk of their current LVO. In this context, in LVOs classified as cardioembolic, initial OAC was prescribed in only 46.8% of the cases, whereas in LVOs caused by large-artery stenosis, PAI and statin therapy at admission were prescribed to only about 40.0%. Although PAI and statin therapy are regular discharge medications for non-cardioembolic infarcts, their low frequency of use in the surveyed collective is unexpected. In particular, 26.0% of cardioembolic, 28.3% of macroangiopathic, and 31.6% of cryptogenic LVOs with a previous history of ischemic stroke neither received PAI nor OAC, suggesting a missed opportunity for secondary stroke prevention. A high-quality meta-analysis reported that long-term aspirin allocation can reduce the risk of ischemic strokes by approximately 20% [18]. In cases of AF, therapeutic anticoagulation with vitamin k antagonists can reduce the risk of recurrent strokes by 42% compared to ASA, or by an additional 19% when comparing warfarin to direct oral anticoagulants (DOACs) [19, 20]. In patients with a recurrent stroke and final diagnosis of cardioembolic LVO, there are presumably cases in which atrial fibrillation was first recorded and thus no indication for an OAC existed so far. However, this raises the question, why 26.0% also did not take a PAI as standard of care.

Among patients with a recurrent stroke, no significant differences were found in baseline characteristics, cardiovascular risk factors, or preexisting AF between patients receiving initial PAI or OAC and those not receiving them. The only difference was a higher rate of concomitant statin treatment in patients receiving initial PAI or OAC. However, patients who were administered pre-admission OAC or PAI showed a trend towards higher mortality during hospital stay (25.0% vs. 11.4%, p = 0.08), although the difference was no longer significant after correction for multiple testing. A possible explanation for this trend could be an increased cardiovascular risk profile or comorbidity in the pretreated group, although differentiation based on the evaluated data was not possible. Increased stroke severity or mortality due to prior OAC or PAI administration has been discussed in the past but has not been confirmed [21, 22].

**Fig. 1:**
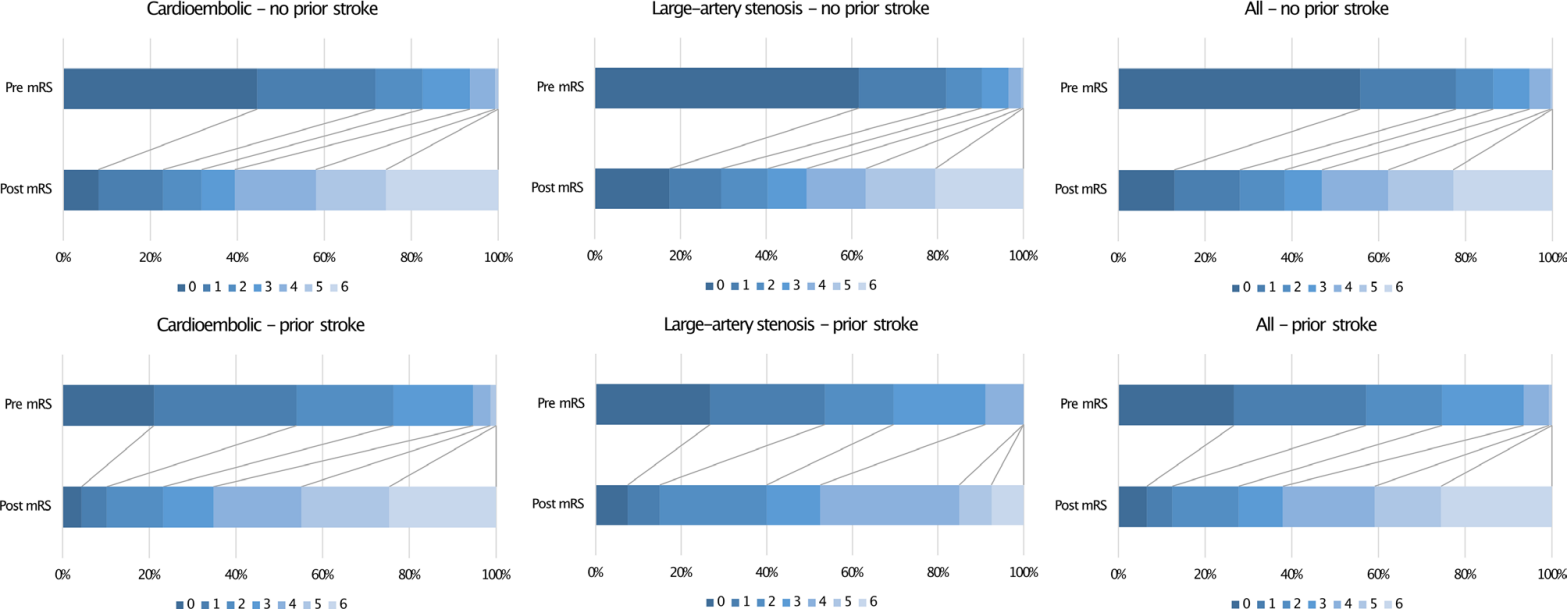
Functional outcome indicated by mRS (modified Rankin Scale) before admission and at discharge between patients with acute LVO due to cardioembolism or large-artery stenosis, subdivided into a positive history of previous ischemic (non-classified) stroke

The data on medication adherence are very heterogeneous, depending on the target group and the origin of study. A study from Singapore showed high medication compliance in only one-third of patients after first ischemic stroke, whereas younger patients or patients without concomitant diseases, such as arterial hypertension, showed poorer compliance [23]. Similar results were obtained from Denmark with an adherence rate to PAI in about 36% and an increased risk of nonadherence in strokes with less severity [24]. *Kronish et al.* reported results in a low-income, minority population in New York, where medication adherence was poor in 40% of cases with more common concerns about their medication, suspicion of their treating physicians, or limited communication with healthcare providers due to language barriers [25]. Comparable studies found non-adherence rates between 38% and 41% for cardiac medication in patients with known AF [26, 28]. A more sufficient adherence rate was reported in up to 85.2% of patients when they were involved in an improved outpatient healthcare delivery system, such as Kaiser Permanente Southern California (KPSC) [27]. However, the reason for the reduced medication adherence in our studied cohort could be evaluated, so it remains unclear whether medication was ever initiated in the past or if there was a specific reason for discontinuation. Nevertheless, our study showed that a significant number of patients with recurrent stroke did not receive appropriate secondary preventive medication, despite nationwide access to the healthcare system in Germany and high standards of quality and guideline-based treatments after stroke [29, 30]. This is particularly relevant given that LVO was associated with a high risk of permanent disability, as evidenced by a shift in mRS at discharge, which was independent of the underlying stroke etiology or stroke history (Fig. 1).

To improve drug adherence, it is necessary to integrate stroke patients into medical follow-up. Moreover, it is essential to provide training to medical and non-medical personnel to enhance drug adherence by imparting specific information to the patients, on the one hand, and by identifying and correcting any incorrect medical prevention, on the other hand [31, 32]. This emphasizes the importance of reviewing secondary prophylactic medication in the context of cardiovascular risk factors and adapting it to current guidelines to effectively prevent the risk of recurrent strokes in such patients. Furthermore, besides drug adherence, it is recommended to conduct a more intensive outpatient search for unaddressed embolic sources, such as atrial fibrillation, in patients with unexplained strokes. Given the limited capacity and associated costs of intensified cardiac rhythm monitoring in the outpatient setting, study inclusion should always be considered in patients with a cryptogenic stroke [33]. Moreover, secondary stroke sequelae such as the occurrence of post-stroke epilepsy should be assessed and treated during follow-up [34].

As this study was a monocentric evaluation, the transferability of the results to the general population must always be assessed. For patients with a recurrent stroke, the evaluation may be biased if the current and previous stroke treatments were performed by the same practitioner, as in-house care deficits may account for the observed effect. However, since the included cohort originated from a large university hospital in a metropolitan region with a wide catchment area where several maximum care hospitals and smaller stroke units exist, the patients were likely treated by different neurologists/hospitals for their first stroke. Therefore, we assume that a representative cross-section of patients was included, and the results are likely to be representative of a high-income country like Germany. In addition, the cohort was from a period immediately prior to the COVID pandemic, so that the results obtained are independent of this and also support a representative conclusion [35].

### Limitations

Limitations of this study include the lack of detailed information about the initial stroke etiology in recurrent stroke patients and their prior preventive strategies. In this regard, it was not possible to determine whether AF in cardioembolic LVOs was detected for the first time during hospitalization or was already preexisting. Additionally, no information was available about the exact indication of the preventive medication (e.g., PAI or OAC) on admission. There were no information about the reasons for the reduced adherence or whether a specific preventive medication had been taken in the past but had been stopped for a certain reason. Therefore, the study only indirectly extrapolated medication adherence and permeation in post-stroke patients based on their intake frequency at presentation, which needs to be further investigated in future studies.

## Conclusions

Although there is general access to the healthcare system and guideline-based preventive strategies after stroke, a high proportion of recurrent stroke patients still receive little to no adequate medical stroke prevention at admission. The reason for this may be medical non-adherence or uncertainty concerning stroke etiology. Given that LVOs are associated with increased disability, improving medication adherence and evaluating unknown stroke causes are essential to maintain an effective secondary stroke prevention strategy.

## Data Availability

The datasets generated and/or analysed during the current study are not publicly available due national data protection but are available from the corresponding author on reasonable request.

## List of Abbreviations

LVO: Large vessel occlusion
IVT: Intravenous thrombolysis
PAI: Platelet aggregation inhibitors
OAC: Oral anticoagulants
mRS: Modified rankin scale
NIHSS: National Institutes of Health Stroke Scale
